# Supplementary material for: Homologous recombination mutants cause differing lethality between h− and h+ Schizosaccharomyces pombe strains due to mat1 heterochromatin
Source: FEBS J. 2026 Mar 23;293(17):5205–20. doi: 10.1111/febs.70507 (PMC13534971; doi:10.1111/febs.70507)
Supplement: Supplementary file 2 [file FEBS-293-5205-s001.docx]

***mat* locus sequence of *h^+N^***

H2, H1, Mat-Pc (3x), Mat-Mc (1x)

AGAGTTGATTGTGTTATTTAAGGAAAAGTGTCATACCCTTTCTAAGTGGGGGATAGCAAAATAAGGAGACAGAAAGTTGGTTGCGTTATTCGAGTAAAATGCCATACCCTTTCCAAATGGGGGAGAGGATTAGGTGAATGAAAAGAACAAATAAGTAGGATTATTCTATTGCAAGTTGTTTGTAATACTTTTTTTGTCATGAACGAATATGTAAAAACAATAATAAATCCTTAGAGCCTACTGTTAATATAATAACATTATGTTTATTCTATGACTACAGGTTTGTCAACATGTTTTATTGCGACTGTTTAATTTTTGTATTTGTTATACCGATATATTTTTAATCAGTATTATATTTTCTTAAATAACTTAATAACGAACTTGACAATGAAACGTTCCTAATTCTCATTAGACGGGAATTCTTTAGGGGACCCCACATTAAGTGGGATGAGTGCTTGCTTTGTTGTAGTAGCTATGGTAACTATTTCTTTGTTTGGGTAGGAACAAAGAAAGAGAAAGGAGAGAAAATAGTGGGTTAGCCGTGAAAGGAAAAGAGAGTGAGAAGAAGGGTAGGAAAAGAAGAGAGAGTAGTTGAAGGAAAAATATTGGAAGAGGTAGTATTTTTCTGTATGCATATGCATATGCATTTGTATAGCATACAACCTTTAATTTAATAGCCATACAACCTAATAGCCAGAAACGCCTTCCCAATTTACTGAACCCTGCTTATATGTAGTTTATAATTGTTGTGTCCATTTAATCTTTTTGAATATAGTATGCGCTCTAACTTGGCAATTTGTCTCCTAACGTCCTGATAATGACTGAGTTCAGCTAATTGTGACCAGGCAAGTCTAACATTTTGTGGTAATGTATGCCAAATTATTGAGCATTTGGCTGAAGCACCTGCCCAATCTCCTGATAGATTCAAAATTTTGTGTAGCCTACTTCTAAATAGCATGAATCCATTTTTGTAGATTGTGGTTTTGTTTCCTTTAATTTGTTTCTTCTTTTGTAAAGTTTCTGGAGTCAAAATTGGCAAGAAAATAGGTGCTCTTAATCTTGGATCCATTTTATTATTGTGCGTATTATGGCTTGGTGTATTTGGAGGAGTGCAACTCTGCTACTGCTGTGGGCGTTTTATATCACTAATGCTTTGTTCCCTCTTTCTTTGTTCCTTATGCGACTTTATATACTCTATGGTTAGTAACACCAATACAATTTCTGAGTTTTATTTTAAGCAATTCATTATGAAACGAGTAGCTGTTTTATTAAAGACTGTAATGTGTGAATTTCTCAAATGTGATTACAATGGATACGATAGAATCATTTCTCTTTTGCGGCGTATTCTTACTCTAATTTGTACTCCAAACTTAAATGGGTTGACTATTAAACGAGTAATTGATTCTATGCAGTCTTTGGAATACATTAAACAAACATGTAATTTCAAACTTCAAATGTGCATTTCCAGTATGGCTTTTAAAAGAAACAATGCTTTACAGAATTGTAATCACTATGCTTGGTGTGATGATCATTGTTCAGATATTGGAAGACCGATGACTACCGTACGCGGTCAGTGTTCCAAGTGCACTAAACCCCACTTGATGCGTTGGCTTTTGTTGCACTATGACAATCCATATCCCTCTAACTCTGAGTTTTACGACCTGTCAGCGGCTACTGGATTAACGCGTACTCAATTACGTAACTGGTTTTCTAACCGACGACGTTGAATTATGTTAGCTTAGAATTGTTAGTAATATATCCAATTCCTTCTTGTATATGTTATACATCAGTTTTAATGGGCCAATTCTACGAAGTTTTAAGGTATTATTCATTCGGTTTTTGTTTTTCATAAATTTTTTTTTTGTAATATAAATGTATAGTCTTTCTCCTTTGTTTTCTCTCGTTCGTTTCCATGGTTTGTGATTATGCTGTTCAGCATTGTAGATCATATCCGTCGAAATCGTTTAAATACTTACATATTCATCAAATTTATTTCATACATTAATTTTAATTGTTGAATTAAAATAAAAATCCCTTTTTGGATACCGATATTTCCGAATACCACGAAGTTATTGGCATCTGTATTTTAAGCAATTAAATGCGATTTGATTTTAAACAGGATTAATAAACGAATTTATAAAGCGTTAATATATTACTCAATTTACCAAAAATAATTATTACCAATTGCTGATGATTCTTATTATAAAAATAAATATTATAGTAAATTTACCTTGTGGTTGATTTCACGTTATTTTGTGCCTATATTTAAAACATTTTAAATATAAAACAAAAGATTAGCAAGAAATATGCTTCGCTTTTAAAAATCATCTAGATTGTCAAACTTAAATCATATAATTTATTTTCAAATTTGAAGTACTGCTATCCCCAAATTGGCTTACGCTTCCTTGTTAGTCCGTTAGTTAACCCGCAGCAATAAACAAGATTAATAGTTCATTTCTATATATAATTTACCAACCGAAAGAGTAAATTTTTT*GGTAGCTTTTAGCATTCCGTAC*AAAAAAAAAAAATGCTGAAATATCGTATATTTTCTAACGGTTTTTTTAGATTACCACAACCATTTGATTTTTTCAGTCCCACATTATTAAATTCAAAGTTTCCTTTGCTTGACTGTCTTCCCTGCGTTGGACTTCAATTAAATTCCATCAAAGATGCGTACTTTTATGCATCAAAATCTCTATGTTTTATATCGGTTGCAAAGGTAACACGGATGGTAGGGTGTATATCTTCAAGTTCTGATAAAAGATAATATCAACATATCTGTGTAATAGCTTGTTTGCCATTTAAGGAAAATCTGATATCTTTATATTCAATTGAAAAAATCTTTAATGAATATTTGGTTTCCCGTCTACTTAACGATAGCTTTTCCTCCAAATCCAGATATAAATTGCGAGATCAGTCTGAACTCCGAAACTGACATTCTTTTGATAAGTCGGTACTTCAATTCAATAAAAGAGTTCATTAAATTGATCGCATAATTTGTTATATTATTTTAAGCAAAATTTCGACTATAAACAGTTATCTATATAAGTTAGTTGCCGCAGGTTGCTGAGATTTTACACGATTTAAAGGTGTTAATTAAAAGAATTGTAGGTTTCTGGAAATATTAACATTTGATAATAAGAGTATCGCAACAGAATAAAAGACGTTAAAAGTACTTCATGTCTAATATTATTTCCTAATACAAATATATTATTAAGCTTGTTGACTTTACTGTATTCTGGACATCTGTCGATCATAAAAACTTTTTGTTGTATGGTATACAACTTCGTGAATCTACATATTATTGTTACGATACCTAAAATAAATTACCTCATACCTTCAGTCTTGATTATGTAAACTTATAATTACGAATCATTAGCTTTACTGGGTCACGAAAGGGAAACGACAATTTTCAGCATTACAAAAACGATCATCAACGATGTTAATCGTTGAATCGCCATTATATACTTAAATTTGAACACCCTGAGTGAGCCAGAAGAAGGAGCAAATTAAGCAAATTTACTCTAAAACATTTTTTTTCGCATCATTGTGATTAATTAATTATGATAAACTGCGAAGACACAATTTACAAACTTTCAAATTGTGTCGGACTTGCCCCACTTTTAAAAAGTTAATAATGCCTATGGAAATCCTTTCCCAATATGAGTTGAATTTCTGTATCTAAGGTATATAGAAGCAGCCTAAGCTTTCATATTAATTCGTTCGAAAGAATCTCTCGGGTGATACAACTTCTTAAACTACTCTACCTATTGCTTTTTGATGACACGGCTAATAAACTCAATAAGTAAAGGTACCGTCACCCTCATCATCAGTTTTAAGCGAAAACGCATTGCGTTGAACATTCATTTTAATGAATTAATTCTTCGTAAATGTATGATTTTCTCGAGTTTCGAAAGTTTTTCCTATTTTCCAAACATACTTTAAACTTCATATAAACTTTGTTGATAGCTATACGGTATCACTAACGAATTTATATACGTTACTTTATTTCACTGTTTTATCTAGCTTTTACTATGAAGTGACGTATAATTTTTTTTTCTTTTTGCAAAAAAAATCAATACATCAAAAAAATACACTGTAAAGTATTTTTAAACATTAAAATACTGTTATGATTGGTTATCGTTGGATGATTTTTATATCCGTTTATAATTATGTCGGGATGTCCATAATTCGTGTTCAAGATTTTTGCACATGCGGAGATAAAACAAAAATAAGCACAATATAAAACTATCTCGTATCCAAAGAAACTAAATGAAATATTTTTTCGAGCATGTTTCGAATAATATATCTTAAACAGAATTCATTAAGGACGATCATATATTACAGAAAAGTGTTTGTAAGCGGCACATCCATTCAATAGTTTGTGATTGGTCATTTTCGATGATTTAATTGAAAGTATAAAACTCAATCTGAAAGTGTCTGTTTTCCGTTCTATTTCGAACTTTTTTCTTTAGCAGTTGTTGGACAGTGGAAATAATTTTTTAAAAAGTTCACAAACGCTTTGAGAATCTCAGAGAAGTATCTAATGCGCGAAGAAAGCACAATGATAAGAACGAGTTTTTGTTGAATGTGAAGGTGGGTTCAATTGACAAAGCTTTTGTGGACGATGGAGGCAAGATTCAATATAGTAATCAAGAAAGATGTTAAGTCAATTCATCGTTGCCTTTATCAGTCTGACTTCGTTCACGCCGTAGCAACTTACTTACATGGAAGTGTAAGCAACGAATGAACTGTGACGCGTTTTGAAAACACAGGTGGAAGACACAAGATGATTGCTACTAGGGACTTGGTATCAACGATATAGGGATACGTTTGGTAGTGCAAAGTAAAATATAAGAATTACGGATTATATATAAAGGGAAAGGATGAAATGAAAGGTATGTAATTGAAGCAAATTTTACGAGAAATATGGCTCAATTGGTTGAATCACATGAAGGATTCTATGAGAAATTTTCATAATAGTAAAGCGATGCGCGCTTGATCCTTTTTCACAAGCAAAATGGATGGCGACAATGTATCCTGCTAACTGTATTTACTGGTCACGATGCTTAGGCGGTGTAATTAGTGAATTAGTCATATCTCCAATTATATAACTTGAAATGGTAACTGTTAGAGACATCGAAACTTGCTATTCCAATTCATTCGCGTAACGATACTTCTTATCTGTCTTCTCCTCCATAACAGCCAGGTAGCGGCAGTATTACTTGTATTTCCAAAACTAGTATTCTGTCGAAATTATCGAAAGCTACATTTTTCCCTGCTTCAGTTTCGCCAACGTGGAAGAAAAGCTTTAACAACGCATAAATATGGCTCGGAAGACATGTCCTCAGCTATCATTGTGGTCACAAACACATATTATATGGATATTTGAACAATGTATAACAAGCTGATAAAAAAAAACAATATAGTGGTGCTGATGGTACCATTCAACTTGACAATCATTAAAGCTCTCTGCAAGCACCCTAAAAAGCAATGAATAGACTCCATTGAGGAATCTGTGCAACTTGAGAGATTCTTTTAAGTGTATATAGATAAATGTTTAAGCTACGCTCTTGCTAACCTTTATACAGACATTCGCGTTTATTTACCGAAAGCAGGTAGTGCTCACATATCTGTTTATTATAAGCGTAAGGCCAAACACAATTTGGTAAACTAGAAAAATATGTATTCATTTCAAGAACCTTGTTTGTTTCGTGGTTTGACAAAGTTCGAACATGCTAGCAGTGATATGACTTGCGCTTCTGATTGCAGTTCACGAACAAGAAAGACAAACTTAAGTCGGGGATTTACTATGTTTGATACATTACATAAATTACGGGATTAGTGCTGCAAATGAATCCTTAGTAAGGATGTGCTTAATCAAGCTTTTAACCTTTAATGTGACGGATCCCATGAACCATTTATTTTACAAGAAGAAAAATAAAAAAGTCGACTTGACCCAATAAAAAGGATTTGAAAATTTTTAGTAAAATATATCATTTCTTTTCAATAAACATCAGCAACAAATCATTAGATACCAAGTTACAAGAAAGGCTCAAAAACTCTTATTCTCCTGTATCACTATATTATCCTATGATGTGCTCTATCTCTCTATTACGATCTTTGAGATTTTTCCTTACTCATTCTTGCTAAATCTTGAAAATACTAATGTTTATTGTCTGTTTTTGAATACGGCTGATAGAATATATTCACAGTCCTAATTCAACTACGTGAATTTAGACACATTGCCCAGTACTTTAAAACTGAAAAATACGAAAGATATGATAATGAAATTTATTTCAGTCTTAATCCTGATATAGAAGAAATAGAGTACTTCTACTATATGAACGCATCATATACTACTCGCATATTGCTGTCTTCTAAACTTTACCAGATTTTAAGAAACTAAGATTTAAATAGAATGAACTATTACAGATTAAAGACAACCCAATCGTAAGCTATTTGATATAATCAGTCATTCTTTTAAAGCAATTGATTAAACTGGAAGAAATACTCAATGAACAGTAGTAGACGGAACAAATGATGTGAGGTTAATGATGGGTTCATCGTTTCCATTCAGTTTACACACCATACGAAGAGGAAAAAGAAGTTGTTAAAAAAGAACAACCGGTGCCGTCAGTTGGATATAAAGAAACGAGAGCTAGTACGTATTACCACACCACCACAATTTAACCCGATTAAGCAACATCAAGAGTCCTGCAAAGACTGAATCAATCGATACATGTCTCGCTGAAAAACTTTATAGTTGAATGTTGTCTCAATTTCTTGTGATATGAGTTTCAAGTTTTGATCTTTTTTAAAATCGTCATCAATCATTTCATCTTCTAGTTTGTTCGTTTTTTTTAATTTTTAAACAATATCGAACTTTAATTACTGGAGTTCAGCCGACTGGAGATGTAAAAGTCTTTATCGTTTTTTGTTGACAATCATACTCGAAAAAAGAAATCAACCCAGAATTGAACACGAAGTATGACCAGAATTGCTCTAGTACACGTAATGTCGTCATGGCAAGAAATTTTGGTGATGTGGGTCTACTAATCCAATAATAGCGACTGCTTACTTATTAAGAGACTTGAAGGCAGTTCAAACATACCTGTGTCTGCTCACATATGCAGCTTCCGGAATCTGTTTTCATCCTACATATTCTTACCCTTCTACTACTCATTCACTTTATGTTTACCCTAGCAAAATGAATTAACATTATTTAAAAAGAATTGGTCGAAAGCATCAAGACTATCAAGCTTAAATACATTCAGTAACGAAATTAGTCATATAAAGTCTTTCGTAATACACAAATTTTTTAGCGCACACGTTATTCCTGCTTAAAGCTCGTTTCTCTTCATCCATTTTACACAGATAGTGTGATCACGAAGAATTACTAATAAAACATTGAGGCGTTATGAGATCTATGTCGCTTCGCAAAACCGTATCTTAGTACGACATAAAGTTGCTTTTGGACTTGAACGTTGACTAAATAAATGTGTTGCCTGATTGTACATGATATATTATCAGCTTTGTCAAGGGAAAAACCGAGACCGAAGAAAGTTAAAACTGAGAGAAATTCCAGATATGCTACTATAAGTGCTCTTTGTCATGAGAGAAACATGATTCTACGTGTCTGATTCACGCAAAGGATGCATCAATGTACCTTCTTTTAATTACAGGATGATGCATTTTTAATTTGTTATTGTTGTGAATTCTATGTCAACTGTTTGCTAATATAGGCGGCGCAGAGATATGAAAACCAAACCGTGACAATACACCTAAGCTAAAAGGTCAACGCACGCCTAAACTAGCAGCTCAACTCATGATTGGACTAATAATTTAAAACTAAAACCTAAACCTATTTTACAAATAAAACTCATAATTAGACTAGCAACCCTACCCATCAACCAACGACTTGACGGTATGCTATTCCTTCTTTTTTTAAGGCTTCACTACCATCGAAATATATATAACAGTAGTAAAATTGTAAACTTTTATTTTTATACCATAGTAGTATGGCTATGAATGGAAGGTTGTAGTGTCAGTCAAGTCGGAAAAACTGTTGGCACTTTTTTTCTGAATCAATGGTATTCTTATCAAACACATGCAAACGTATAAAGAAGACTTGAATAGATAATCTTTGCATTCAAACAAATCCTAGTCAACTGAACAACGCATCTACCTCAGCAGTTCTTGGGAAATGTATAATAGGCAAGCATTAAACTTTTATATATAAAACCATCGAGATCTCTGTTTTTAACTCGGTGGTTTCTTTCACAAGACTTGCTTCAAGGACGACAACACATCCTGACAGTTAATTCATTAAATACTAATACTATTGAGGATTCTGTTATCCAAGTGGAATGAACATGATCATCAAAGGAATGTTTCCTCAAAGTGAGAACTATAGTGTTGCAAGAGCGAATACTTGGTTGCTTAGAACAAAACGAATTACTGTCATTAGAATATGCTCAAAAGTGTGACGCTATATCTTGTATCTATTGCTACGTTCATTCATTGATCCAACAGCATGGGTATAGAAAAACGATAAGCAGGAGTTGCGCAAACGAAGTTATGGAGTTTCTTGCATGCATGCATGTTTTGGCATAAAGCTGGTACTTCGAAATATGCCTATGAAATCTCTACCTGCTTTGCACCCTAAATATAACGTGTCGTCTTGTAGCTGCATGTGAATTACAACGATTGATAGATGAAAAACAAATCGACTTGTTGTCCATTTCATCGTTTCAAACGACTGGTAGTGTTGCTTGTGTCCATATCCATACTATGTCCGTGTGTTTTGCTTTATTACACTTGGTCAAAGAAATCTATATGATGTTCTTTACGAAACCCACAAAGTCATTAGGGATATCATCCGAACAGAAGGGTATATATGCAAAATACACTGCAAGGACTAAGCCCAAGCACCGTATTGTTTCAGTCTCGAAGTAATTCTATGTACCCGTGCATTGGACATGGTGCGATTGGACTTGAGTGAGCTGACAGCACTGTATGAGGGTCTGTTTGCCAAGATTCAGGACTTGTTGAAAGAGTTATGTTTTGATACAGATGTGGAAAAACCATTATCCATTTCGTTGCTAAGATCGATTGGTGACGACGATATTATCAATAGCAAACTGGGATATTCATTCTTTAAGGATTGTATGGAGATACGTTTTTCGAAAAGTGTATTGTTACGAATACCAGTGTATAAGAACAGTGAACTTTATCTTTGGTTTTTATCGTCGATGTCTAAGAAGGATCTAAGTAATTTGTTTGAGAAAACAGATCAGCAAAACAAACAATTGCTACAGTAACTCCAATATTAGCAAGAGTTATTACAACTACAACTATAACTATAACTGCAACTATAACTACAACGATAAGAACTATCACTACTCTGAAGACAACGATGTGTTTTCTCAAGATGATAACGGATCTAGCTTCGCCATCAATAAGTATGAGACAAAGGAGTCCATCTTTGATGAAACATCCATTTGCCTGTTGTACATTTTTGCAGGACAACCAGCCTGAGCACAAGAGACATGGTGTACTAGACTTTGTGCGGAATGTCTACTTCAAAACTCGCATCTTTATATCTGATATATAGAGAGAGGCTGATGATTTATAAACACAAACGATAAGACTTGTAACATGAAGTTTGTCGAAAAAGTCAATCTTTAAATTCCTTTCTGAACCTCTCTGTTATGTCAGTGCTTCGGTATTATGCTTTAATGCAGTCACTTGAGGCGTTGTCCTAATAGGTGCAAAGTTACCGTATATTTAGGTTTCATGTTTGTGATTACTGGAGAACGACTGTGAAGAGACCAACCGTATCGTGTTTTTTCCAATTGTGATCTTCCAATGCTTAATAAATACGAAATCTTATTTCGATTTACAGGCTGGGCATTCACGAAACATAGCGTTTTTACATTTCTACAGGCACACCATAAACAACTTGCAATATCTGCCATCACTTTATTTCTCCAACTTTGTTCAAGTTAAAAGTGGCAGAAAGTGTTTCCCGATATGAGATAACCCAACGCATATGTTGTTGCAAAAACTAAACGCCCATTCATCAAACGAGTCGAGATGAGAGGAATCATTGTATAAAAGGCTAGCATGTAAAAGCTGGAAGAAGGAAAGAACACGGGATATGTAATGGAACATGGGACCTCTTGATATGGACATTGATATGAACATGAATATGGTATAGAGAACTACATCGACACAGAAAAGAAAACAAAAGTTGCTAAAATGACAAAGGTGCCGAATCAACAGGCCATAGTACCGATGGAATCCAAATACAATCCAAGAATCATTCAAACATCGCAAACATCTTTGCTCGACATTGTTGTTTTGAACAACTACTAAGAGTTGCAAATGTGAAAGCAACAACATCCAACGTATCTACAAATTTCTCGCTGTTTTGCATATACATTTATATTTAATAGTGAATTAGCGTATAAGAAAGAAATGGAGCTTTTTTGGATTACAAGTGATAAGAGTAGGTGTAGGAGTAGGACTCATTTATTGAATCTGGTGATTTGCGTCGGAAGGTTGAGAATATAAAGAGCGTGTGAAACAAGCCATGTAATCTAAAACTTTACCGAAACTTTCAGATCTCGCCTATTTACCGATCCAATTGCTAGTGCAGTAATAGTTGCTTGAATTTTCACCGGATGAATCTTCTCTTGAATAAAACCACCAATAAGAGCTAGTCAAGTTGACTATTAAAAACACACAATATTGAAGATGGCGGTTGGTGGAAGCTTTCCTCTCAGCATTCAGTATTACACAAACGGATTAAGGAGAAGCGGTAAACGACTTTGAGGCTCATTTATTGTAACGATTTTTACAGTCTTTCTTTTTTCTTGTACGATAATTATGTACCATTTTGCTTAATTCTCTTTATTTAAGAAAAGAATCTACTTTCCTCTGATTCTTCGTACATGGGTCAATTAATGTATAAGCAAAACCTCGGAAGAGTACAATATGACTTCCAATGCGAAAGAAATGGTATGAAAATGAACTATATACGATTATTGTTTTTTTAGTGACGGGCAAAATCATTATCGAGTGGAGTGACTTTTCACAATCCCTGTAATTCAGTTGAGATTGTACTGTACTGTATTTAAAGTAGGTGCATATTTTCATTTTTTAAATTATATTTGATAATGGGTTGTTTTTTATTTTCTAGCTAAATCGAAGGATTAAACAGTATCTTCCACATACGTCATCAATTCTATAATAGAAGTTTTACCTAATTAAACAGCAAACCATTTAAATAAACTCATATTTTTCGTGGCTAGTCTGCCAAATTCGTTGACTTATATAGATATGAAGGGTACATATAAAAAATATGAGATTCGAAACGGAGTTAGTATGTGGAACAAGAGAAGTAGATACTGTTTTGTTCTTATATTTCTATAGTCCGAGGCAATACAACTTTGGCGATCATTTACTTTTGTTTTTTTATGAGGAGCAATTTGGCAAATCAATTTGGGCAATCACATTAATCCCTAAAGTATTGGCTTGGAAAAGAACAGGTATGTTTTACTCTGTAAATATCTCCTACTGTCAACCTTCAAAACACACAACATTAAATAGTAATGTGTTACGTAAAAAAATAGTAGAGAAATGCAATTAACAACACTAAGCATACCCGGTGTCTTATACATCAGAAAGATATTTAAACCACTATCGCAAATCCCAAATTTCGCAAATATTCACTAAAAGCTCATTTACTGCCTGGTAGCTAACTATCGAACAGTTAAATAAAATTTGTACTGTTTTCGACTTGATGTAAAAAAGATTGGATAAAGTGAAGAGAACCCTACAACAAGACTGGAAAATCAAAGTGCGCTAAACAAACGTTTTCCGTACCTTAAAATGTAAGCAGGCGAGGAAGCAAAATGTTTGAATAGCGTAACTGGGGTTTCTGAAAATACAAAAATAGAGGAAATGCCCAATAGATCATTCTTGCTAAAATTATAAATAAATATAATGAAATACACAATTTCAAAAACAAAACATCAAAAACCAGGCAAGAAATGGTCAACAAGATTTTAACATCCCCCAAATGGAATGGCTATCCAGTCTTCACACACAATATGCATTTTAATAGAAAAAAAAACATATGTATATTTTGATCGCATAATTGACAAACACAAAATAGTATACAATCAAACAACATCTATTAAGGCGTCTTTCAGATGAGCATCAGCTAAAGAAATATTACTCACAAAGTGAATGGCCGACCACATATCAACGGCACAACAGTATATTGAAATCAAAACGCAATTCAAACAACATACCACCTCATCAGCTAAAACTAGAATTACATCCGCTCTCGCCAAACCTAAAAACTTGATGAAACAAGAATCTTAAAACTAATAAATAAGACGGACTTCAACTAATTACGCAAGTGATTTCAAGTAGTTTTAAGACTCATATATTTGTAAGTCACGTTTGAGAAATCAAATCTGCGTTCTCAAACAATAACGTAAAACACAAGAAAAAACACTTAAAAAAGTAAGACAAATATAGAAAAAAAATTTTTTTATCATATCAATTACAAACATATTGTTTGCTGCAAAACAAAACTAAACATAGGTGAAGTATCCAATGACGTAATTTGAAGTACAAAAGGAGTAACAAGTTACTTATAAAAATAGAAAAACAAAATGGATTATTACAAACTTAAATAGGATTAAACTTAAACTAATTACACCAATCAACTTAACATGAAGCAACTCCTGATACTTTACAGGCCGCCGCACGCGCACCTTAACAGGTGCTTGCTTGGCAGCCTCGTAGGCTTCTAAAAGTTATTTTTCTGTATGCATATGCATATGCATTTGTATAGCATACAACCTTTAATTTAATAGCCATACAACCTAATAGCCAGAAACGCCTTCCCAATTTACTGAACCCTGCTTATATGTAGTTTATAATTGTTGTGTCCATTTAATCTTTTTGAATATAGTATGCGCTCTAACTTGGCAATTTGTCTCCTAACGTCCTGATAATGACTGAGTTCAGCTAATTGTGACCAGGCAAGTCTAACATTTTGTGGTAATGTATGCCAAATTATTGAGCATTTGGCTGAAGCACCTGCCCAATCTCCTGATAGATTCAAAATTTTGTGTAGCCTACTTCTAAATAGCATGAATCCATTTTTGTAGATTGTGGTTTTGTTTCCTTTAATTTGTTTCTTCTTTTGTAAAGTTTCTGGAGTCAAAATTGGCAAGAAAATAGGTGCTCTTAATCTTGGATCCATTTTATTATTGTGCGTATTATGGCTTGGTGTATTTGGAGGAGTGCAACTCTGCTACTGCTGTGGGCGTTTTATATCACTAATGCTTTGTTCCCTCTTTCTTTGTTCCTTATGCGACTTTATATACTCTATGGTTAGTAACACCAATACAATTTCTGAGTTTTATTTTAAGCAATTCATTATGAAACGAGTAGCTGTTTTATTAAAGACTGTAATGTGTGAATTTCTCAAATGTGATTACAATGGATACGATAGAATCATTTCTCTTTTGCGGCGTATTCTTACTCTAATTTGTACTCCAAACTTAAATGGGTTGACTATTAAACGAGTAATTGATTCTATGCAGTCTTTGGAATACATTAAACAAACATGTAATTTCAAACTTCAAATGTGCATTTCCAGTATGGCTTTTAAAAGAAACAATGCTTTACAGAATTGTAATCACTATGCTTGGTGTGATGATCATTGTTCAGATATTGGAAGACCGATGACTACCGTACGCGGTCAGTGTTCCAAGTGCACTAAACCCCACTTGATGCGTTGGCTTTTGTTGCACTATGACAATCCATATCCCTCTAACTCTGAGTTTTACGACCTGTCAGCGGCTACTGGATTAACGCGTACTCAATTACGTAACTGGTTTTCTAACCGACGACGTTGAATTATGTTAGCTTAGAATTGTTAGTAATATATCCAATTCCTTCTTGTATATGTTATACATCAGTTTTAATGGGCCAATTCTACGAAGTTTTAAGGTATTATTCATTCGGTTTTTGTTTTTCATAAATTTTTTTTTTGTAATATAAATGTATAGTCTTTCTCCTTTGTTTTCTCTCGTTCGTTTCCATGTTTCCAATTATGCTGTTCGTGTCATTCATTCTCCCTCCAATTTTCCCTTTT*TGTGTGCGCCCTTCTACCTT*CCCCTTCCCTCTAACGAGATATTTGCTTCGCTACGCTACGCACTCCCTACCATAATATACTCACTAATATTATACTTTCACTACATCTCATTACTCATTCCTACCATACACACTATACTTCAACACTTTCCTCGCTCAACCACTCCATCAACCATCCTCTTTCTCCCCATACATCTTGAACTCCTTACACCAATACTTCATATCCATTAGCCATTTTCCTTTATAAACATATATTAAACCTCATCCCAAATGCATCTACCCACTCTTTACATTCACATCCCTATTCCTTTAATATCTTACGTTCCACTTCCTCAACCATAGAGCATTCTTTATAAATCACTTTAATATGCTTTACTTCCACACACTACTGCTACCATTGCCTTGCACAGTACTCCCCTTACTCACACACCTTCAATCACACTCCTATCTCCATACAGGACCCAACATGTATTAAACATTATCCCCCTACACCATTTTCCCTCATCATCCAATATGTTTCTACTATTATCACACACCGTCTCTACCTCCCGCCAATTTTAACTGTCTTCATTTCCACCTACAACTCTCTATCCACACTTTCTCACCCTATAAACATTTAATACACTCCTACAATATACCATCCCATCCTATTATCATATATACTCCTCCTTCTCAGATCACATCCTCTCCAACTCCATTCACACATCTTTTTCTTTACACACCCTCACCCACGCTCTCCATACCTCACGCAAACCAATATACACATACCCAACTCACACTAAAAAATTAAATTTTACCTATCTATTAAAGATATTTACCCTGTGTTGTACCTAATCCATCTACAACACCCGCATAATCATTATTCCTATTCCTTACTAACATCATTACCCCTCCACTAAATCACAAAATTGACACTTTTTTAAAAAATTTGCATCAAAAAACAGACCACAATTCTTCACTTTCCTTAACAATCAACCCTTTTCACTGTATATTGACTACTTTTCCATTTCTTTCACAATAAAAAACACAAATACTGTTTTTTTCTCTCAAATCATCACCAACCACTCTTCTCAAAAATATTATTTCTACAATGGCACAGCTCACATCAAAATCTTCTTTACATCCTTCCTTTACACGCAAGCAACCTTCAAACCTTCCTCCTACACGCCAATAACCATGACCCTTCGCTATCATCCCGTGCAAACCTTTTAAATCCCTACAAACACTGCACAAAAATCACATTTAAACTTATACCAATACTCTCGTACCCATTCTATATACGATCAAAATCGCCTCTCCACACAACTTTTTGCTTGCAAAAAACTCCTCTCAAACAAAACATCAACATCATATATCAACTTTTCACTCTAACTTTTAAATATTTTATATTCCTCTATCACCTTCCATATCAATTACTTTACCACTTTTTATAATGAAGATTGAAACATGCAACAAATAATTCAACTTCCTACATCTCTTAACCAATTTCAGACTTACTCATAAAAACACAGACCGCCTCCTTAAACAGCAACCTTACGGTAAACTCACGTTTTGCTATTTACTACTATAAACTAAAGGCTTCGAATTCATAATTCCGTTTAGTGTGCTACAACGAAATGCTTTATAATAATAAAATAACATAGTAAGCATCACTCATAATACATACAGCTACTAATAATAAAATATCTATCCACTTTAAAAAAAAAGGTCGCCAGACTTAAATACCGAAAAGTAATTGAATATTTAAATTTATAATTCTAATGTTTTTAATATTTATCAAATACAATAACTACGAAACTATCTTATTAATTTTCGAAAAACATATGAATTACTTCATTTCTTTAACTCTAATTAAATCTACCAACAATTTTGTCGTCCTTACACTAAATCAGTCATTAAAATAAAAAAAAATATTCTATAAAATATAATCAGCTATACCATTTTTAGTTTTCACCATACAACACCATAATGTTTCTTTATTCCCTAAATACACTTCAAAACGACAATGTATTGATTATCTGACCTTAAAAAAATTTATTAAAATTTTCATTAATTTTATAATCATTGTATTACTTTACTATCAATTTTTTTAAATATTTATAACCTATTTTCTTAATAAATATTTAGGTTTAAATTCTAGTTGCCCATTATAAATCAAAAGCAATCACTACTACAGAACGTTTCAATGAGACATGCTATTAACATCGAATCACATTCAAACGAAAACTAACACAATACTAAAAATTTTGTAACTGCTTTTTTCTTTCAATTCTATTATAACATTGTTTATTTATAAACCATACTCAATTTTGTAACCCTTCTAACGCCGAATGACAAAATACGCTATTTGTTTCATCGTAATCAACCAAACTACATCTTCTCATTTACACCGCAAAGCTATAATTAACTTTATTCAGTCAACTGCAAATAATTACCAACTTAAGCTACTTTACAAAACATGTTCCCAAAATCAACATGTTTTACATTTCACACAAAATATAAATATTTTACGAACATTTAAAGCTACTATAATCCAATTCATCAAAATGGTAAAACTACCATACTTTTAAAATATAACAGACTAATTTAATACGAAAATATTTACCTCATTAATTTCAAGCAGACGTTTTTTATCCTCTACATCTATCACAAAATAATACATGTAAATATATATACACTATTCATAGTTCCATCATAGAACCAAACATAACTTTTATTAACTTACTACACTTATTCTCAACGCAAACATAGCTAAACGACTGCTCATATAACATATAAACTATTGTAATTTGACATTCAACGTAACTTTCTGTGTCCCTTAACTGCATAGTCATACTGAACTGGCCATGCAACATTTTCACCCAACTGGTTCGCGCTGTCTAGCACCAACGACGGGTTCCTTAAGAACTCCCTTGCGACAAAAGTAACATCAGCTTTTCCACTTTCTAGGATTTCATTCGCCGTAAGACCATCCCTGATCATTCCGACAGCGCCAAGTAGTATTCCATTCACTTGATCCTTAATCTTTTCCGCAAAAGGAACTTGATAGCAATCCTTCACCTCAATTCTTTGATCCTTGTGATTACCACCAGAGCTAACATCTATCAAGTCAACACCACCATCGCGAAGCCTCGCTGCTAATGCAACTGTATCTTCTATCTCCCATCCTTGTCCTTTGGGCAACCAATCTGTAGCCGTTACTCTATAAAACAAGGGCATACTATCTGGAATTGCTTTACGAACAGAATGGACAACTTCCATAGGAAACAAAATACGTTTCTCAAATGTCCCACCATACTTGTCATTGCGGTCATTAGTGGCAGGACTAACTGTTGACGATATAAGATAACCATGAGCGCCATGAATTTCAATTACATCAAAACCTATTTCAACTGCACGCTTCGCAGCAACAACAAACTTATCCACTAATTCATCATATTGCTTTTCAGTTAACTTATGAGGTTGAGCGTGGTTTTCGTCCCACCTGTCTTCATTTGGTCCATAAACATCATTCTCCCACCCACCTTGAGCTTCAGTCGCAACTGTGTATCCTCGATAAGGAGCAGTGGTGCTAGCCTTTCTACCAGCATGCGCCAATTGAATCCCAATTTTTTGATTTTGCGAATGAGCAAATTCAACAATTCTTCGTAACGGCTTCATTTGCGACTCCATCCATAATCCTGAATCATTAGGTGAAATTCGTCCCTCTGGGGAAACCGCTGTCGCTTCTACCATTACAAGGCCAGGCCCACGCATTCCCATCGCTCCAAGATGTACTAGGTGAAAATCTGTCAAATGCCCTTCTTGGTCAGCGGAATAAGTGCACATCGGCGAAACAAACATTCTGTTATGGAACTCCACTCCTCTAATTTTAAGAGGAGTAAATAATTTTGTTTGCGGTACACCTATCGCCGCTCCAGCTGGTATTTTTTGCGCAGGTGTAAAATAACCAACATTTTCGGCTCCTTCATTAACAATAGTCATTTTTAAAATTTTTCAATTCAAATTAATATATTGGCCAGTCGTTGGGACATATTTATACACAAAGCATAATGTTATTCCTTACTAAGCTAACCGCAATATTTCCGTGCAACAAACCATGATCATATTACATAATATCTTTTCATCATTCTATTCGATAGCACGGCAACATTGCAAATTACAGAAAATTTAACAATGAGGTTTCAAATAGAAAGCTGTCGAAGTAAAAGTATCATTTGTTTCTTTAATCTACCTCAACAAAGAAAAGAACTATAATAGGAAACCTGTTAAAGATTACGAATGCTTCAATATAGCGCTTTTTGAATTCCAACTAAAAAATATTACAAAAATCAAATGATTAACAAGCTGTCATAATCAAAATACTAATAATGTTGAAATATCCATTGCTAAGTTAAATTGCTCAAACATTACGAGAATCGATTTATTGCATACATTAGTAAATAGTCATTCTAAAAAGCAGCAGCAATTCTTCATACTGTATATGTGTATTATTGCAATTTTTTTTTGAAATATATACAAACTTACCAATTGCCAAAATACTTAACCCTTATTTCTAAAATTAAACTATAATAATATTCATAACAAGCATATTAAACACCATCGACTCACAATATTGCTAGTTAAATAAGATTAACTATTCTTTTTTTAATCAACAAAAATATACCAAGACCTATTTAAAATCTAAAACTGGCTTAAGCATATCAAAGATGCAAAGCAATCTATAAGTTACGTAATAAGTATAAAGGCAAAAAACTTCAGAAGCGTGAAAAACACATCCCATTCCAAGCGCATACACTCAAAAGTAATACAAAAATGTCAAAAGATTCGGACTACAAAAGGGCTGAAAAGCATTTATCTTCAATTGATAACAAATGGTCGTCTCTTGTCAAAAAAGTTGGACCTTGTACACTTACTCCACATCCAGAGCACGCACCCTACGAAGGAATTATTCGAGCTATAACATCACAGAAGCTTTCAGACGCTGCCACAAATTCAATTATCAATAAATTTTGTACACAGTGCTCAGACAATGACGAGTTTCCTACTCCTAAGCAAATTATGGAAACAGACGTTGAAACTTTGCATGAATGTGGCTTTTCAAAATTAAAGTCTCAAGAGATACACATTGTTGCAGAGGCTGCATTAAACAAGCAAATACCTTCTAAATCGGAAATTGAGAAGATGTCGGAAGAAGAACTCATGGAGTCACTAAGTAAGATAAAAGGAGTAAAACGATGGACCATAGAAATGTACTCTATCTTTACACTCGGACGACTGGACATAATGCCTGCGGATGACTCTACTCTGAAGAATGAAGCAAAAGAATTCTTTGGATTATCTAGCAAACCGCAAACTGAAGAAGTTGAAAAACTAACTAAACCTTGTAAACCATACAGAACAATAGCTGCTTGGTATTTATGGCAAATTCCGAAACTCCATCGTAAAGGCCAGTAAAAGTCGTTCAAATATCTAATGGTTCCCGCTTTTGTTTATGAACATATAATAAGCCATACTTTATCTAGTACACATGCCAAACATAAAGTTACTGATTAATTTTTCTAATCTTTCTTAATATTTATCACTAACTAATACAAATCAATTAGTAGATGTGTATACGTACGATCGAAAACCCGCAATTTCATTGTAAACATAGCACCGGTCTAGTTACCGCGAACAACAAGAGCTGCGTTGACTGCTTTTTTTTTGCTATATAATCCAGATGCAGATTATTTTAAAATACTAATCCAAATATTTTGCCTGCCAGCACTCCATAATTTAGTTTACGAGTGGTGGTATACTGTAATACTTGGTATACAAAGAGTCGACTCCACCCTGGATTAACTTTTCCTTATGTATATTCAGGACTAAATCAACGACTGACAAACAACCCATTAATCGATATAGAATTTTTAACGAATAGTAAAGGATAGAATTAATTTTGAAAAGAACGTTTATCTACAATCTAAATATGTAATATATAATAACCAACAATCAATTAAGTACAAACAATTTGACTAAACTTATAATCATATTCATTAATACATATGTAAAATCATCTACTTGAAAAGCTTTTGTAAACTCATTTCTCCAGAATCGCCCTTGTTTAATACTTTAGCAACAGCTAATTCAAAATCTTCTTGAGTAACATGAACACGTCTTTCTCTCAATGCAAACATACCGGCTTCAGTACAAACACCTTTTAGCTCGGCACCACTAGCACCGTTCATTTTCTCCGCTATACTCTTCAAATCAATGCCACGTGTTAAATTCATGGACCTCGAATGGATTCTCAAAATTTCGGCACGTGCCTCAGCAGATGGAGGTGGGAATTCAATTTTACGATCAATACGACCTGGACGAAGAAGAGCAGGATCCAAGATGTCGATACGGTTAGTGGCCATAATGACCTTGATATTTTTGGTTGCTTCAAATCCATCAAGCTGATTCAACAGCTCTAACATGGTCCTCTGAACTTCGCTATCGCCACTTCCACCACTAGAATCAGATCGTGAAGAGCCGATGGAGTCGATCTCATCCATGAAAATAATAGAAGGAGCATGCTCACGAGCCATAACAAATAGTTCACGAACCATACGACTACCTTCGCCAATATACTTTTGAACAAGTTCTGATCCACTGACACGGATGAATTTGCAATCGGTATGGTGGGCAACTGCTCTAGCTAACAAGGTTTTACCGGTTCCCGGGGGACCATAAAGAAGAATACCTTTAGGCTGAGGAATACCTAAGCTTTCAAAAAGTTCTGGATGCTTCACTGGTAATTCGATAACTTCTTTAATTTCTTTGATTTGCTTTTCAAGACCACCAACCATCTCATAAGTGGAGTCAGGAATTTTTTCGACCATCATGAGAGAGACAAGTGGATCAACCTTGTTGGGGAGGATTTTAATTAATTGGTATGAATCGTTCCTCAAGGCTACACGAATATTAGGTTTAATCTCCTTAATATCAATATCTGGACTAATATCAACGACATATTTGCCTTCAGGATGGACTTTGACTAGAACTTTGTTCTTTCCCATTGTCTTAATCACTTCACCGACATAACTTCCGGGTTCCTGAAGCAACTGAATCTCCTCTCTTAGAAGGCGAACTCTTGCATTTAGTCCGTTTCTTTGAGCCTCTAATCGTCGTAAATTTTGTGTCTTTTGGAGAATAGCAAGTTCAGCATCTTGAATTTTTTGGGTATAATACTACATTTTATGTTAACAATTAATTAAATAACTATTACATACCTGAACTATGTTCTCATTACTCTGTAAAACGTTAGTCTTCAGCACCTCCGTCATTTTATACTACTTCAGCTTCTATGGTGGTTCTTCGTAGAAGATTGCTACCAGCTACAAATAACTATGCAGCAGTATTACGAGTTCGCTGGGTTTCGTTATTTTTATATGCTTTTGTTTAGTAATTATGAATAGTTATTGACACGAAAGGGAATGATGATTATGCCTTGTTGTTGTTACAAAATTTAACTAATCATTCTTTATTTACTTTTTCATATACAATTGAGGAATCCCCCGCCTAATAAACAAAAGTATTTTGCCTAAAATTTTATATTATACCAATATAAACAGACAACTCAATAAATATGTACCTTATACATTGAGGAACAGTTAAGCCATTCCCAAGTGCATTATTTTTAGCTTCAAGGAAAATAGAAAAGACTGTAATTGAGGTCTCGGGAAAAAGACATCCCAGATAATGCAAAAGTGGTTAAGCAAAGCTAACTCGCAAAAATAAAATGACCCATTAATACCAGATTTTAGACACTTTCAATGTCTTTTTTTTCAAAATTAACTTTCTCCTTCGAACCAGAGCCAATCGATATGGTTTCCACCACAACGCCTTCGTCTTCAACAATATCATTAACCTTTCTTCCGAATATTGAAGCTGCCATGTTGCTTACAACTTGATAAATATCATTACGTCCTTCCCACAATGGGAAAATTCCAACAGCGAATACGGTAAAGAAAATCCATATTAATCCAACGACTACCCATCCTGTGAAAAATCTTTTGGAAAATATATATCCAGTTCCATACATTGGCCAAGGCCAAAGAATAATAAATGCTGCAGTAATGATTAATGCTGCAACCTTAGAGATAACACTTGCACGATTTAATTTCTTTGTTTGTTGAATCTCATCTTCTTCAGATGAGAAATGCATTGCAAACGTGGCCAACAAACGATTGAAATCGTATTTTTCCGGAAAAATTATTGACAAAATTAAAATATATAATGCTGGACTCAATAAACCAACAACGTTACCAGCAAGCATTGGGTTGTTTGCACCAGTCGTCTCAATAGTGAGCGTTTTGCCTCCCTCAGCACGAGCAACAACAAGCCAAGTAATAATACTCGAAATGATTCCTAAAACCGGAGAAACGGTAACTGCAATTGTAGACACGCGGCTAAAAAGCATTACACAGGTAGCTGGAACAACAGCTGGACATACTAAAACACCCATCAATAGATAAAGATATCCCATTGATACTTGTCCATAATAAAGTCCAGTGGCAAAGCCTGACATTGCGAAGCCAAAAACTATCAAAGATGCATGCCCTGTATATAACAATTCCTTGCCGGTGGCTCTGGGTCTAACGTATTGCTTGTAAATATCATAGGTAAAGATTGAAGAGACGGCTATTAATTCGGCAGACGCTGCAGATGTAACAGCCATGAAAACAAGCAATAAAGTAGCGTTTGCGCCAGCTCGACCCATCAATGCGATAGCGGCATAAGGCAAAACCAATCCTTCACTAACTTCCAAGTCACTCATTCTGTTGGGATAAGTAGGGAAATAGGGTTTATTTTCTAAACCGAGAGCCACGAGACCCATGGTTGTGGCAGCAAGCCAGGGAATTGCAAACCATGCAAGACCACCAAGGATATAGCCAGGTAAAGCAGATGCAGGATTAGCAGCAATAGCTTTTTGCCAATATCCATTATCTACGAAAACTGTTCCAAAGTTTCCTGCCAAATTGATGATAAAAAAGATAGCACCCTCTTGGCTTCTCATAGTTAAGTAACTACCTTGAGCGTTTCCAGCTACTGGATGAGCATCACCTGCTTCCTTTAACATATCATATAACTTTCCAGGAGATCCGATTTTTTTATCAGCAGAATAAGTGGCCAAGCTAAACATGATCAAAATGACCAGGATAATAACGGTGTGGATATAATCAGTAAGGAAAGTGGCTTTAATACCTCCAAACATAGTATAAATAATAACACCAACTGGTAGCAAAAAGCAAACAGCTACAGTGTTCATGCCCGTCACCGAAGAAATTGTTGCCGATCCACCACACAACAACATGGCCATAACAAGTATATTAGTGATATATGCAAAAACCAGAAATACACCGTGTGCGATGGGGCCACATCGAGCTCTGACAACCTCAAGGAATGTATGGGCGTTAGGAGCTTTGCGCTTGAGTTCAATTGCTAGTACTGTAAACAGCAAAATTTGGACGCATGCACCTGATGCATACCAAAACGCACCTGATACACCGTACTGGTAAGTTTTTTGAGCACTGGTCAACAGGGTAGAAGCCCACGTCCAACTACTAACTACTGCGCTAGCAACCAATCCAGTCCTTACGCTATGCGAGGCAGTGTTAAAATGCTCTGATGTTTGATTTTCGTTATTGAACTTTTTCAAACTCCATGAAACAAATATCATGAGTGCGGCAAATCCCAAGCCAAGGCCCACAACGATGCCATAGCCAACACTTTGCGTCAATTCGGGTTGGACCATGTTGTGACAATGTTGGTGATTACTTTTGCTAACAGATCTAAGGTAATGAATAGATGGTTAGAGAATTTTTTTCTCTACTTGTCACTCAATCAAACCTATAAATCTTGAACTTTTTAAATCATTCTTAACTGCTCTATTTATAATACACATCTTTCCGTAATTTATGAAAATTTACTAATTCCAATTATCAAACAATGTTGTGATAAATGATGCTGATCTCATGTACCATCTCGATATAACAATACTGTATATCGTTTAGCAAATAAGATAGCTGATAAGTGACAACAACCGTAAAGGAAAAGACTATTTCCTCTCGCTAAAGACTTATCACACCAACACTATTGATCTCTATCAGTTAAGCTAAATTAGTAATTATAAACAAATTTGAAACTTGTATAACAGAGCTAATGCTTGCTCTATTTGAGCAATAAATCGATTGCTTTTCCTACAGCATACTCTTTATTTTTTTTTTTCAAAATAATACGTAAAAAAAATTAATAGTCACTGTAAACTAGCAAATCGACGTTATCACGGAGTAAAACGTCAATATCACTTTTCTCCTAGATCTTTATCTTCGTAAACGAAGTTAGCGAAGTGTTGTCGATGCAATCATTGACACTAAAGTTGGGGACGAGACTGTGAATCTCCAAACAATCACGCCTTACAAACATTTCAAAGTTTCGTTTTCTTTTTTTTTTGGGATTTTTTTTTCGGCTACATGTAATAGCTAATGAAAAAATAATATTAACCAGTAAGATATTTGTAAACAAACTACATGCAATCCTCCAGATAGTGAGATATTTGAATTATTTACCTACGAAAAAGAACGACTCTATTATGCATATTAAATAGGCCAAGCTTGCCCTTTCTCAACTTGAATTACGCTGGTAGAACATTAAAACAGACTAAATTTGCAGCAAATGGACAGGTTTCGTAGAATTTGAAATCACGATATTTGATCAACACGGAGTGAATGCGGGATGGCATAAAGTGCAGGGAAAAGAAAAAATATATCAGGAGACTGAGCAACCAGCATACTTTCTCCTGACAACTAACCCTATCTTATTTTTCTTTTATCTTAAGGAATCTTAAGGGGCAGATTTGAATGTGTTTACAGTATTCTAGTATAGCAAAGGTACTACTAGTTCTTCCGATATCACAATCTTACGGTATTTGGTAATAACGATATCTTTCTGCGTACGATAAAATTCGTGTTGACGTCTTGTTAGCTTACGCCTGTCAGAATATTCCAACAAACGGCATTTAAGACAAACTATGTTCTCTTAGATTAACAGACTAATTAGAAGTACATTAGATAATATCTTTTTTATTAGTAGCATTTTGTCGAAGATAGCTAGCATTTCTTGAGTTTACGCTAATGTCGAAGGAAGATGAAGAAGTCTTAGAGTCGTACTTTTGGACACTTCAAGAGTCTATTCCATTAAGGAGAAGGATTCGAGAAATTCGAGAACTACAGGAAAGGAATAAAAACAAATACGAACAGTTATTGCAGGCGAGGAAGGATTTGGACCGTTTTCGAAGTAATTTGAACGTGCAACAAGAGCAACTGCAAAACGAAATACTTGGTTTCAAACAAGACGTTAAGCTTTTAAACAATGTCTTGGTCTCTTATTCTTCTGAAGAACTTGAAAAGAGATTTGAAGAGTCACTTTTGAAAGAAAAAGCTACGAGTATGGAGATGGAATCTAAACTTTGCTCAAAATCCATAACTCCTTTGGAATATGTTTCTTGGCTTTTGAATTCCATTGAATGAATGAGCGAAGGAACATCTTGGGTAATCGAATCACTCGACTCATTTTTTTTTCCTTATCGATCGAGATAGTATGCGAATCCTATTTCATTTCACTACTCAATTGTGTCGTGGCAATAAAGATATCATAGTTGACACGGTGATTAATTTTTCCCGGAACTCTGGTAAAATTCTCGAATGAATTGTTTCATAAAGGGAACCAAATACTTTTGAAAGAACTTACTCTTGCACATGCAACTCAGTGTGCTTACTACATTTTCGTTTTTATGACGATTAGTTAACGTTTGTAGTAGATTACCCGATACGGTGCTGGATAAGAACCCAAATTTAATTCATTCTTAAGTTAGCCGGGTGTCCTTAAAAGTTTGAATATTGGGATTCTATTAATCCTACATAAACCGTTAAAATTAAACAGTTTGAAAAGCTTTTCATATGTCGACATGAAAATTTTATGACTATTCAGTGTCTATTAAATAAACACAGATCGGTCAGATTTAAAAGTTCCACTTTTGTTCTCTATAAACATTTTTTTAAAATGGGGGAACAGTTCAATTATTCAAATGGCCTCTAGCTTAAAAATAGGTTTATGGTCTCATTGAACATTTTAGTTTTTAACTATTACTGTTGATTGTGAAATTCTTTTCATTTTGACATCGACGCGAAACAAATACAAACGATTTATTGCTTTGAGGTCGTATTTAAGTTAGTAAACTTTGGTATCTTGTAGTGTCTGAAACCTTTGACATCGAACACATTTCTTTCATTTTACACGGCGGATTTTCTTCTATTTTGCCAAACTTTGTTAAGCCAGGAAGGTAATCGATACTTGGAATTATTACATTTTAAATACTCATCTATTAGAAACGTACATAAACAGTAACCAAAGCTAAAATGACTAGGAAACTTCTTAAAACTTGGTTTGAGTATGCTACTTAACTTCCACTACTATATCTCCCATCATCAATATACAAATGTTGAATCAATGCATTAACATGAAATATCGTGCCTAATCAAAAAGACCGAATCCCATCTAAATTAGTTAGTATATATCATTTAAAATCGCATATAAAACTTACATCTTCATCAGATTCCTCGGCTGCTTCTTCCTCCTTAGCTTCTTCGGCAGCGGATGTGGCCTCAGCGCCAGCAGCACCGCCAGCAGCAGGGGTGGCTACAGCACCAGCAGAAGGGACAGTGCTGAGTTTTTCATTGCCTGCAGCAATTAGCTCTTCAATGTTCTTTCCGTTCAACTCGCTGATTAAGCTTTCAACGCGTTCCGCTTCAGCTTCAATTCCAACCGTTGATAAAACACTTTCAATATCGGATGCTGAAGGTGACTGCTTTCCACCAACAGTAAGGAGTAAATATGCAGCTAAATATTTCATATTGACGGAACAAAATCACTTTAAGCAAAGAAAATAGAGCTTTGCAGAAATTGTCTCAGTTTCCAAAATTTTTGCTGTTGCCCATTTATATAGGGCTTTTGAAAGTCATAACCCTACCCTACTTTATATAAGACTAAAATTCTTTCAAATGAGCTTCCTTAACGATGATCGTAAAAATAAACATAAGTGTTCGTATAATAAATTTTTATTAAAGCTTTAGTTTCTTTGGAATTAAATGAGGAATTTCTGAACATGATCACATCTTAAATCATCAAGGTTAAAATATCGCTTGTTTCCCATAGCAATTCACAAAGTAAAGACGATTACGAAAGTATTAATAACTCAATGCTATCGTAAAAAAATAGAAAATTTTCAAATGACCTTTTTATTTTTACGTAGTTTTGCTAAATGATTCTTTTTCTTATAATTTAATTTAATAGTCTGAGTTATTTGTTCGTTAAATATCGGTGTGCGGCATAAACGTGGCATCTAATTTTTGAGTTATTTCTTTACAAATAAAAAAGTTGTACAAATAAAATCAATATAAATCATATGTCCTTCGGTTTTTTTTTTGCCAAGCGAAAATTCCATATTTTGGGACAATTGTGCTTGCAAATCTTTCAAAATAATATGATCTTTTTTTAAATTTCATTTCGGGGTTATGTACCCACTTGTTGAATTAATAATTTATTCTTTCTTTAGATACAATGCTATATTTTGAAGCGTGACACAACAAATCTTATATTAAATTTTCAAACAAAAAAGACAGACAAACAAGTTTTCGTGAAGAGCTATTGAAAATTCGGGTAAAATAATTTAAAAAAGAAAAAGAAAGAAAGATGAAACTATCAGAAAAATAGCATTTATCTGAAACAACTAAATTAAAATATGCTTAGTGCGATTATTAATTACGGTTTAAAGTGTAGATTGCGACTAAACGAGTATTATTCGTAAAATAACATTTCGTTGTACAATAATCCCTAATTTGTATATAACGTAATGTAAGGTTCTACTTTATCATTGAGCAGCGGTTCGATTTGCGACCCTTAACTATTATGTAAATAAATCCCGGTTCGAGTCCTATAAATGCCTATAATTTACAAGTTCGCAGATGAGCTGATGGATAAATAGTATAGCTCTTTCGATTATAGAAAGCCATTCACCCTAAACATCCTGAGTTCGCATGAATTATATCCTCTATTTAAATGTCTTATAATGCTGCTACGAACAGCTTTAGGGTTTTTAATGTATAGTGGGGGTTTTTTTTTTAACAGGTATGTTAAAAAGAATTAAATATTTGTTGTGATGGTTTTATTTAAGGATCTTCTTACCCACAGCCTGAAATTGTTAAGTTTACCTAACTAAAGCATTAAAAAATATCGCTGTATAGGCATCCTTTACCAAATTACTTTGCCCAATCTTCCTTTTATATCTATTCTTTATTGATGGAATATTACGAATCAAACACAGATGTAGGTGCTTTAACAGTTTATCATGATCATCGGTTGTCTTTTTTAATACATAACTGCAACTCAGCATGTGGAACAGGAGTCACTCATCTACGGTCTAGTTACGATGTCATAATATATAAGCATAAAATTACTGGTGTCTAAACAACCTAACTTGATTTTTTAAATTCTACTACAATATTTCAATTTTAACTATCAGGTATGATTTTAAATTTTATATCATAATACAATTATCAGGTATTTGAGAGAAACAGCCAGTGCGTTTTGATGTTGCATCATACTGGTTATTTTGCATTCAATCTGTCTTGAGCTCAACGTTGGTATAATTAGAGCTTCTGAGTTTATATTTTGCAAAATAGACATTGGCTTCAAACAAAACAAAGAAGTTTGGTTTGTAAATCCGTAAAAATTGCATTGGTATGTGTTGATCGTGAGTTTGGGGTTTGCCGAGAAACTCAATTCTCTCGACAAATAAATGTGAATTTGAGCAAAGGCTACAAAGTTTCTGTAAGCATTCTTCGATAAGTCACTTAGACGGTTCGTACTGGTGCCCAGGACCCCAAACCATCAATTGACTTGTCCCTGCTTTAGAAGTGTCAGGGTCATCGCCATTACGTCTTTACCTACTCATATTTTTAGCAATTATTCCAATTCATCTTAAAAATTTCAGCCAACATTATCTAGTCAGAAATCATATAGAAGTAATATGTTTTATATTCGCATATACATTTGTATAACTCTTGACTTTTTTTAATAAGCAACGTTGCGAAAATAAGTATAAATTCTTTAGCTAGTAGATCAAAAAATTGGATTGTTTTCATATGCTTCCATCTATATTCTATTATTTAAGAATGCAGATGAATTCATCATTACCATGTTAATGTAAAAGAGCTGGCCACCATCTTATAACCTTTTCTTTAAAATTTTTATAAGTATAATAGTCGCTCAGGAAAAAGGAAGAACGGCAAATAATAGAAAAAGGTGGAATTTCTTAAATTTGTAATTAAAGTAATTTCTATTCTTTTGTATGAAAAATCAAATCACCGCTTTTGAGATTAATCGTTTACATCTGGTTTTTTAAGAAACTGATTATAAATCCAATCGTTAAACTTAAAAGATCTTTGCTTTTAGAAAAAGCGCAAATACAATTTTATGAGAACCCGACGATACAATATATAGACTATGGGTTGATTATAAAAAGCGGAAGGAAGTATTGATCTACATAACTTACTATGTCATTACCGTTGTGTAAAAGTAGGCAAAAGTTTAAAATGGAAGCAAAGCTGGACAATAAGTGTTGATGCGGTATTTTGGAAATAGAAAAACAAAATTATAATTCAGAACAACCTTCTCATAAAAATCTGTTCTACTCATACCAATCCATTAAAAAGCCTAAAAAATACCTAGACAAATGACCAAGATACGCTACTAACTTTATAATAATATTTCGCAAGCCGTCTATTGACTTTCTCACTTTCAATTTCACACAACATTTAAATACAAATGTACATTTACAAATATAGTAATATGCTGGTATGGACATAGCAATTTAGCACTATATTAGCCTTTATTTAACAAAAATCACTCTTTAAATAAACAGTTCTAATAAAAAATTCAATTAAACCCATTCAAAAACTAACTTTACAGTACATGAATAAAATCTTAAGATTATCAAAAAAACTTTCAAACATGTTCAAAGTTTACAACCATGTAGGCATCGAGAAAGCTGACAAACAAAAATGAATACATCACGTTCACTTCCCAATAGTTATATAGTTATACATATTTCTTAACAATTTAACCCTCTTAGCTTTCTACATTTCCAAACATCATATTAAACCTACTCGAATCCATATGACTACAACTACCTTCTAAAAGTTTCAAAACAGTTTGAAAAGTTTCACCAAGGCAAACAATTATCTTTTGAAATACAACATTTCAATATGTCAAATCCTCAAAAACATACAGCAAAAAAATGCCAACATGATTAAAATAAAATTTTAAAATACCCAAAAAAAATCAACCAGTAGGACATTTTTATAGTTCCCAAATACGCATCTCCACTTAAAAGAATAAAAGGAATTTATATTCCAACCTAAACTCAGCAAAAAAATTCATTATGATATAAAACATAAAAACAAAAACAATCACCAAAAATTATAAAGAAAACCAAATCTATGATTGCAAAATTCTAAAAAAGACATAAATCGCAATAAAAAGAACAAACTAATTAACTAATAATAATAATAATAATAATAAAACAAAAAAGGAAGAACTAACAACATTTTCAATTCCTCAACATAAATGAATTCCATTTAAACCACAATTATTTCATAAATAATCAAAAAAATATATATATGAATACCACATTAACAAGTAGGAAAACAAATAACATGTTCCTTCGCCTACGTAAGTTTACAAACCGGACAAAATTATAAACTAAATAAAACTAAAAAAAAAAAAGTACACAATTATAAGGACCCATACAACAACGGATTACTAAAAACAGCAGTTTAAATGTTTTAGTTCTGCCGCGCGCGCACCTTAAACAGGTGCTTGCTTGGCAGCCTCGTAGGCTTTTAAAAGTTATTTTTCTGTATGCATATGCATATGCATTTGTATAGCATACAACCTTTAATTTAATAGCCATACAACCTAATAGCCAGAAACGCCTTCCCAATTTACTGAACCCTGCTTATATGTAGTTTATAATTGTTGTGTCCATTTAATCTTTTTGAATATAGTATGCGCTCTAACTTGGCAATTTGTCTCCTAACGTCCTGATAATGACTGAGTTCAGCTAATTGTGACCAGGCAAGTCTAACATTTTGTGGTAATGTATGCCAAATTATTGAGCATTTGGCTGAAGCACCTGCCCAATCTCCTGATAGATTCAAAATTTTGTGTAGCCTACTTCTAAATAGCATGAATCCATTTTTGTAGATTGTGGTTTTGTTTCCTTTAATTTGTTTCTTCTTTTGTAAAGTTTCTGGAGTCAAAATTGGCAAGAAAATAGGTGCTCTTAATCTTGGATCCATTTTATTATTGTGCGTATTATGGCTTGGTGTATTTGGAGGAGTGCAACTCTGCTACTGCTGTGGGCGTTTTATATCACTAATGCTTTGTTCCCTCTTTCTTTGTTCCTTATGCGACTTTATATACTCTATGGTTAGTAACACCAATACAATTTCTGAGTTTTATTTTAAGCAATTCATTATGAAACGAGTAGCTGTTTTATTAAAGACTGTAATGTGTGAATTTCTCAAATGTGATTACAATGGATACGATAGAATCATTTCTCTTTTGCGGCGTATTCTTACTCTAATTTGTACTCCAAACTTAAATGGGTTGACTATTAAACGAGTAATTGATTCTATGCAGTCTTTGGAATACATTAAACAAACATGTAATTTCAAACTTCAAATGTGCATTTCCAGTATGGCTTTTAAAAGAAACAATGCTTTACAGAATTGTAATCACTATGCTTGGTGTGATGATCATTGTTCAGATATTGGAAGACCGATGACTACCGTACGCGGTCAGTGTTCCAAGTGCACTAAACCCCACTTGATGCGTTGGCTTTTGTTGCACTATGACAATCCATATCCCTCTAACTCTGAGTTTTACGACCTGTCAGCGGCTACTGGATTAACGCGTACTCAATTACGTAACTGGTTTTCTAACCGACGACGTTGAATTATGTTAGCTTAGAATTGTTAGTAATATATCCAATTCCTTCTTGTATATGTTATACATCAGTTTTAATGGGCCAATTCTACGAAGTTTTAAGGTATTATTCATTCGGTTTTTGTTTTTCATAAATTTTTTTTTTGTAATATAAATGTATAGTCTTTCTCCTTTGTTTTCTCTCGTTCGTTTCCATGGTTTGTGATTATGCTGTTCAGCATTGTAGATCATATCCGTCGAAATCGTTTAAATACTTACATATTCATCAAATTTATTTCATACATTAATTTTAATTGTTGAATTAAAATAAAAATCCCTTTTTGGATACCGATATTTCCGAATACCACGAAGTTATTGGCATCTGTATTTTAAGCAATTAAATGCGATTTGATTTTAAACAGGATTAATAAACGAATTTATAAAGCGTTAATATATTACTCAATTTACCAAAAATAATTATTACCAATTGCTGATGATTCTTATTATAAAAATAAATATTATAGTAAATTTACCTTGTGGTTGATTTCACGTTATTTTGTGCCTATATTTAAAACATTTTAAATATAAAACAAAAGATTAGCAAGAAATATGCTTCGCTTTTAAAAATCATCTAGATTGTCAAACTTAAATCATATAATTTATTTTCAAATTTGAAGTACTGCTATCCCCAAATTGGCTTACGCTTCCTTGTTAGTCCGTTAGTTAACCCGCAGCAATAAACAAGATTAATAGTTCATTTCTATATATAATTTACCAACCGAAAGAGTAAATTTTTT*GGTAGCTTTTAGCATTCCGTAC*AAAAAAAAAAAATGCTGAAATATCGTATATTTTCTAACGGTTTTTTTAGATTACCACAACCATTTGATTTTTTCAGTCCCACATTATTAAATTCAAAGTTTCCTTTGCTTGACTGTCTTCCCTGCGTTGGACTTCAATTAAATTCCATCAAAGATGCGTACTTTTATGCATCAAAATCTCTATGTTTTATATCGGTTGCAAAGGTAACACGGATGGTAGGGTGTATATCTTCAAGTTCTGATAAAAGATAATATCAACATATCTGTGTAATAGCTTGTTTGCCATTTAAGGAAAATCTGATATCTTTATATTCAATTGAAAAAATCTTTAATGAATATTTGGTTTCCCGTCTACTTAACGATAGCTTTTCCTCCAAATCCAGATATAAATTGCGAGATCAGTCTGAACTCCGAAACTGACATTCTTTTGATAAGTCGGTACTTCAATTCAATAAAAGAGTTCATTAAATTGATCGCATAATTTGTTATATTATTTTAAGCAAAATTTCGACTATAAACAGTTATCTATATAAGTTAGTTGCCGCAGGTTGCTGAGATTTTACACGATTTAAAGGTGTTAATTAAAAGAATTGTAGGTTTCTGGAAATATTAACATTTGATAATAAGAGTATCGCAACAGAATAAAAGACGTTAAAAGTACTTCATGTCTAATATTATTTCCTAATACAAATATATTATTAAGCTTGTTGACTTTACTGTATTCTGGACATCTGTCGATCATAAAAACTTTTTGTTGTATGGTATACAACTTCGTGAATCTACATATTATTGTTACGATACCTAAAATAAATTACCTCATACCTTCAGTCTTGATTATGTAAACTTATAATTACGAATCATTAGCTTTACTGGGTCACGAAAGGGAAACGACAATTTTCAGCATTACAAAAACGATCATCAACGATGTTAATCGTTGAATCGCCATTATATACTTAAATTTGAACACCCTGAGTGAGCCAGAAGAAGGAGCAAATTAAGCAAATTTACTCTAAAACATTTTTTTTCGCATCATTGTGATTAATTAATTATGATAAACTGCGAAGACACAATTTACAAACTTTCAAATTGTGTCGGACTTGCCCCACTTTTAAAAAGTTAATAATGCCTATGGAAATCCTTTCCCAATATGAGTTGAATTTCTGTATCTAAGGTATATAGAAGCAGCCTAAGCTTTCATATTAATTCGTTCGAAAGAATCTCTCGGGTGATACAACTTCTTAAACTACTCTACCTATTGCTTTTTGATGACACGGCTAATAAACTCAATAAGTAAAGGTACCGTCACCCTCATCATCAGTTTTAAGCGAAAACGCATTGCGTTGAACATTCATTTTAATGAATTAATTCTTCGTAAATGTATGATTTTCTCGAGTTTCGAAAGTTTTTCCTATTTTCCAAACATACTTTAAACTTCATATAAACTTTGTTGATAGCTATACGGTATCACTAACGAATTTATATACGTTACTTTATTTCACTGTTTTATCTAGCTTTTACTATGAAGTGACGTATAATTTTTTTTTCTTTTTGCAAAAAAAATCAATACATCAAAAAAATACACTGTAAAGTATTTTTAAACATTAAAATACTGTTATGATTGGTTATCGTTGGATGATTTTTATATCCGTTTATAATTATGTCGGGATGTCCATAATTCGTGTTCAAGATTTTTGCACATGCGGAGATAAAACAAAAATAAGCACAATATAAAACTATCTCGTATCCAAAGAAACTAAATGAAATATTTTTTCGAGCATGTTTCGAATAATATATCTTAAACAGAATTCATTAAGGACGATCATATATTACAGAAAAGTGTTTGTAAGCGGCACATCCATTCAATAGTTTGTGATTGGTCATTTTCGATGATTTAATTGAAAGTATAAAACTCAATCTGAAAGTGTCTGTTTTCCGTTCTATTTCGAACTTTTTTCTTTAGCAGTTGTTGGACAGTGGAAATAATTTTTTAAAAAGTTCACAAACGCTTTGAGAATCTCAGAGAAGTATCTAATGCGCGAAGAAAGCACAATGATAAGAACGAGTTTTTGTTGAATGTGAAGGTGGGTTCAATTGACAAAGCTTTTGTGGACGATGGAGGCAAGATTCAATATAGTAATCAAGAAAGATGTTAAGTCAATTCATCGTTGCCTTTATCAGTCTGACTTCGTTCACGCCGTAGCAACTTACTTACATGGAAGTGTAAGCAACGAATGAACTGTGACGCGTTTTGAAAACACAGGTGGAAGACACAAGATGATTGCTACTAGGGACTTGGTATCAACGATATAGGGATACGTTTGGTAGTGCAAAGTAAAATATAAGAATTACGGATTATATATAAAGGGAAAGGATGAAATGAAAGGTATGTAATTGAAGCAAATTTTACGAGAAATATGGCTCAATTGGTTGAATCACATGAAGGATTCTATGAGAAATTTTCATAATAGTAAAGCGATGCGCGCTTGATCCTTTTTCACAAGCAAAATGGATGGCGACAATGTATCCTGCTAACTGTATTTACTGGTCACGATGCTTAGGCGGTGTAATTAGTGAATTAGTCATATCTCCAATTATATAACTTGAAATGGTAACTGTTAGAGACATCGAAACTTGCTATTCCAATTCATTCGCGTAACGATACTTCTTATCTGTCTTCTCCTCCATAACAGCCAGGTAGCGGCAGTATTACTTGTATTTCCAAAACTAGTATTCTGTCGAAATTATCGAAAGCTACATTTTTCCCTGCTTCAGTTTCGCCAACGTGGAAGAAAAGCTTTAACAACGCATAAATATGGCTCGGAAGACATGTCCTCAGCTATCATTGTGGTCACAAACACATATTATATGGATATTTGAACAATGTATAACAAGCTGATAAAAAAAAACAATATAGTGGTGCTGATGGTACCATTCAACTTGACAATCATTAAAGCTCTCTGCAAGCACCCTAAAAAGCAATGAATAGACTCCATTGAGGAATCTGTGCAACTTGAGAGATTCTTTTAAGTGTATATAGATAAATGTTTAAGCTACGCTCTTGCTAACCTTTATACAGACATTCGCGTTTATTTACCGAAAGCAGGTAGTGCTCACATATCTGTTTATTATAAGCGTAAGGCCAAACACAATTTGGTAAACTAGAAAAATATGTATTCATTTCAAGAACCTTGTTTGTTTCGTGGTTTGACAAAGTTCGAACATGCTAGCAGTGATATGACTTGCGCTTCTGATTGCAGTTCACGAACAAGAAAGACAAACTTAAGTCGGGGATTTACTATGTTTGATACATTACATAAATTACGGGATTAGTGCTGCAAATGAATCCTTAGTAAGGATGTGCTTAATCAAGCTTTTAACCTTTAATGTGACGGATCCCATGAACCATTTATTTTACAAGAAGAAAAATAAAAAAGTCGACTTGACCCAATAAAAAGGATTTGAAAATTTTTAGTAAAATATATCATTTCTTTTCAATAAACATCAGCAACAAATCATTAGATACCAAGTTACAAGAAAGGCTCAAAAACTCTTATTCTCCTGTATCACTATATTATCCTATGATGTGCTCTATCTCTCTATTACGATCTTTGAGATTTTTCCTTACTCATTCTTGCTAAATCTTGAAAATACTAATGTTTATTGTCTGTTTTTGAATACGGCTGATAGAATATATTCACAGTCCTAATTCAACTACGTGAATTTAGACACATTGCCCAGTACTTTAAAACTGAAAAATACGAAAGATATGATAATGAAATTTATTTCAGTCTTAATCCTGATATAGAAGAAATAGAGTACTTCTACTATATGAACGCATCATATACTACTCGCATATTGCTGTCTTCTAAACTTTACCAGATTTTAAGAAACTAAGATTTAAATAGAATGAACTATTACAGATTAAAGACAACCCAATCGTAAGCTATTTGATATAATCAGTCATTCTTTTAAAGCAATTGATTAAACTGGAAGAAATACTCAATGAACAGTAGTAGACGGAACAAATGATGTGAGGTTAATGATGGGTTCATCGTTTCCATTCAGTTTACACACCATACGAAGAGGAAAAAGAAGTTGTTAAAAAAGAACAACCGGTGCCGTCAGTTGGATATAAAGAAACGAGAGCTAGTACGTATTACCACACCACCACAATTTAACCCGATTAAGCAACATCAAGAGTCCTGCAAAGACTGAATCAATCGATACATGTCTCGCTGAAAAACTTTATAGTTGAATGTTGTCTCAATTTCTTGTGATATGAGTTTCAAGTTTTGATCTTTTTTAAAATCGTCATCAATCATTTCATCTTCTAGTTTGTTCGTTTTTTTTAATTTTTAAACAATATCGAACTTTAATTACTGGAGTTCAGCCGACTGGAGATGTAAAAGTCTTTATCGTTTTTTGTTGACAATCATACTCGAAAAAAGAAATCAACCCAGAATTGAACACGAAGTATGACCAGAATTGCTCTAGTACACGTAATGTCGTCATGGCAAGAAATTTTGGTGATGTGGGTCTACTAATCCAATAATAGCGACTGCTTACTTATTAAGAGACTTGAAGGCAGTTCAAACATACCTGTGTCTGCTCACATATGCAGCTTCCGGAATCTGTTTTCATCCTACATATTCTTACCCTTCTACTACTCATTCACTTTATGTTTACCCTAGCAAAATGAATTAACATTATTTAAAAAGAATTGGTCGAAAGCATCAAGACTATCAAGCTTAAATACATTCAGTAACGAAATTAGTCATATAAAGTCTTTCGTAATACACAAATTTTTTAGCGCACACGTTATTCCTGCTTAAAGCTCGTTTCTCTTCATCCATTTTACACAGATAGTGTGATCACGAAGAATTACTAATAAAACATTGAGGCGTTATGAGATCTATGTCGCTTCGCAAAACCGTATCTTAGTACGACATAAAGTTGCTTTTGGACTTGAACGTTGACTAAATAAATGTGTTGCCTGATTGTACATGATATATTATCAGCTTTGTCAAGGGAAAAACCGAGACCGAAGAAAGTTAAAACTGAGAGAAATTCCAGATATGCTACTATAAGTGCTCTTTGTCATGAGAGAAACATGATTCTACGTGTCTGATTCACGCAAAGGATGCATCAATGTACCTTCTTTTAATTACAGGATGATGCATTTTTAATTTGTTATTGTTGTGAATTCTATGTCAACTGTTTGCTAATATAGGCGGCGCAGAGATATGAAAACCAAACCGTGACAATACACCTAAGCTAAAAGGTCAACGCACGCCTAAACTAGCAGCTCAACTCATGATTGGACTAATAATTTAAAACTAAAACCTAAACCTATTTTACAAATAAAACTCATAATTAGACTAGCAACCCTACCCATCAACCAACGACTTGACGGTATGCTATTCCTTCTTTTTTTAAGGCTTCACTACCATCGAAATATATATAACAGTAGTAAAATTGTAAACTTTTATTTTTATACCATAGTAGTATGGCTATGAATGGAAGGTTGTAGTGTCAGTCAAGTCGGAAAAACTGTTGGCACTTTTTTTCTGAATCAATGGTATTCTTATCAAACACATGCAAACGTATAAAGAAGACTTGAATAGATAATCTTTGCATTCAAACAAATCCTAGTCAACTGAACAACGCATCTACCTCAGCAGTTCTTGGGAAATGTATAATAGGCAAGCATTAAACTTTTATATATAAAACCATCGAGATCTCTGTTTTTAACTCGGTGGTTTCTTTCACAAGACTTGCTTCAAGGACGACAACACATCCTGACAGTTAATTCATTAAATACTAATACTATTGAGGATTCTGTTATCCAAGTGGAATGAACATGATCATCAAAGGAATGTTTCCTCAAAGTGAGAACTATAGTGTTGCAAGAGCGAATACTTGGTTGCTTAGAACAAAACGAATTACTGTCATTAGAATATGCTCAAAAGTGTGACGCTATATCTTGTATCTATTGCTACGTTCATTCATTGATCCAACAGCATGGGTATAGAAAAACGATAAGCAGGAGTTGCGCAAACGAAGTTATGGAGTTTCTTGCATGCATGCATGTTTTGGCATAAAGCTGGTACTTCGAAATATGCCTATGAAATCTCTACCTGCTTTGCACCCTAAATATAACGTGTCGTCTTGTAGCTGCATGTGAATTACAACGATTGATAGATGAAAAACAAATCGACTTGTTGTCCATTTCATCGTTTCAAACGACTGGTAGTGTTGCTTGTGTCCATATCCATACTATGTCCGTGTGTTTTGCTTTATTACACTTGGTCAAAGAAATCTATATGATGTTCTTTACGAAACCCACAAAGTCATTAGGGATATCATCCGAACAGAAGGGTATATATGCAAAATACACTGCAAGGACTAAGCCCAAGCACCGTATTGTTTCAGTCTCGAAGTAATTCTATGTACCCGTGCATTGGACATGGTGCGATTGGACTTGAGTGAGCTGACAGCACTGTATGAGGGTCTGTTTGCCAAGATTCAGGACTTGTTGAAAGAGTTATGTTTTGATACAGATGTGGAAAAACCATTATCCATTTCGTTGCTAAGATCGATTGGTGACGACGATATTATCAATAGCAAACTGGGATATTCATTCTTTAAGGATTGTATGGAGATACGTTTTTCGAAAAGTGTATTGTTACGAATACCAGTGTATAAGAACAGTGAACTTTATCTTTGGTTTTTATCGTCGATGTCTAAGAAGGATCTAAGTAATTTGTTTGAGAAAACAGATCAGCAAAACAAACAATTGCTACAGTAACTCCAATATTAGCAAGAGTTATTACAACTACAACTATAACTATAACTGCAACTATAACTACAACGATAAGAACTATCACTACTCTGAAGACAACGATGTGTTTTCTCAAGATGATAACGGATCTAGCTTCGCCATCAATAAGTATGAGACAAAGGAGTCCATCTTTGATGAAACATCCATTTGCCTGTTGTACATTTTTGCAGGACAACCAGCCTGAGCACAAGAGACATGGTGTACTAGACTTTGTGCGGAATGTCTACTTCAAAACTCGCATCTTTATATCTGATATATAGAGAGAGGCTGATGATTTATAAACACAAACGATAAGACTTGTAACATGAAGTTTGTCGAAAAAGTCAATCTTTAAATTCCTTTCTGAACCTCTCTGTTATGTCAGTGCTTCGGTATTATGCTTTAATGCAGTCACTTGAGGCGTTGTCCTAATAGGTGCAAAGTTACCGTATATTTAGGTTTCATGTTTGTGATTACTGGAGAACGACTGTGAAGAGACCAACCGTATCGTGTTTTTTCCAATTGTGATCTTCCAATGCTTAATAAATACGAAATCTTATTTCGATTTACAGGCTGGGCATTCACGAAACATAGCGTTTTTACATTTCTACAGGCACACCATAAACAACTTGCAATATCTGCCATCACTTTATTTCTCCAACTTTGTTCAAGTTAAAAGTGGCAGAAAGTGTTTCCCGATATGAGATAACCCAACGCATATGTTGTTGCAAAAACTAAACGCCCATTCATCAAACGAGTCGAGATGAGAGGAATCATTGTATAAAAGGCTAGCATGTAAAAGCTGGAAGAAGGAAAGAACACGGGATATGTAATGGAACATGGGACCTCTTGATATGGACATTGATATGAACATGAATATGGTATAGAGAACTACATCGACACAGAAAAGAAAACAAAAGTTGCTAAAATGACAAAGGTGCCGAATCAACAGGCCATAGTACCGATGGAATCCAAATACAATCCAAGAATCATTCAAACATCGCAAACATCTTTGCTCGACATTGTTGTTTTGAACAACTACTAAGAGTTGCAAATGTGAAAGCAACAACATCCAACGTATCTACAAATTTCTCGCTGTTTTGCATATACATTTATATTTAATAGTGAATTAGCGTATAAGAAAGAAATGGAGCTTTTTTGGATTACAAGTGATAAGAGTAGGTGTAGGAGTAGGACTCATTTATTGAATCTGGTGATTTGCGTCGGAAGGTTGAGAATATAAAGAGCGTGTGAAACAAGCCATGTAATCTAAAACTTTACCGAAACTTTCAGATCTCGCCTATTTACCGATCCAATTGCTAGTGCAGTAATAGTTGCTTGAATTTTCACCGGATGAATCTTCTCTTGAATAAAACCACCAATAAGAGCTAGTCAAGTTGACTATTAAAAACACACAATATTGAAGATGGCGGTTGGTGGAAGCTTTCCTCTCAGCATTCAGTATTACACAAACGGATTAAGGAGAAGCGGTAAACGACTTTGAGGCTCATTTATTGTAACGATTTTTACAGTCTTTCTTTTTTCTTGTACGATAATTATGTACCATTTTGCTTAATTCTCTTTATTTAAGAAAAGAATCTACTTTCCTCTGATTCTTCGTACATGGGTCAATTAATGTATAAGCAAAACCTCGGAAGAGTACAATATGACTTCCAATGCGAAAGAAATGGTATGAAAATGAACTATATACGATTATTGTTTTTTTAGTGACGGGCAAAATCATTATCGAGTGGAGTGACTTTTCACAATCCCTGTAATTCAGTTGAGATTGTACTGTACTGTATTTAAAGTAGGTGCATATTTTCATTTTTTAAATTATATTTGATAATGGGTTGTTTTTTATTTTCTAGCTAAATCGAAGGATTAAACAGTATCTTCCACATACGTCATCAATTCTATAATAGAAGTTTTACCTAATTAAACAGCAAACCATTTAAATAAACTCATATTTTTCGTGGCTAGTCTGCCAAATTCGTTGACTTATATAGATATGAAGGGTACATATAAAAAATATGAGATTCGAAACGGAGTTAGTATGTGGAACAAGAGAAGTAGATACTGTTTTGTTCTTATATTTCTATAGTCCGAGGCAATACAACTTTGGCGATCATTTACTTTTGTTTTTTTATGAGGAGCAATTTGGCAAATCAATTTGGGCAATCACATTAATCCCTAAAGTATTGGCTTGGAAAAGAACAGGTATGTTTTACTCTGTAAATATCTCCTACTGTCAACCTTCAAAACACACAACATTAAATAGTAATGTGTTACGTAAAAAAATAGTAGAGAAATGCAATTAACAACACTAAGCATACCCGGTGTCTTATACATCAGAAAGATATTTAAACCACTATCGCAAATCCCAAATTTCGCAAATATTCACTAAAAGCTCATTTACTGCCTGGTAGCTAACTATCGAACAGTTAAATAAAATTTGTACTGTTTTCGACTTGATGTAAAAAAGATTGGATAAAGTGAAGAGAACCCTACAACAAGACTGGAAAATCAAAGTGCGCTAAACAAACGTTTTCCGTACCTTAAAATGTAAGCAGGCGAGGAAGCAAAATGTTTGAATAGCGTAACTGGGGTTTCTGAAAATACAAAAATAGAGGAAATGCCCAATAGATCATTCTTGCTAAAATTATAAATAAATATAATGAAATACACAATTTCAAAAACAAAACATCAAAAACCAGGCAAGAAATGGTCAACAAGATTTTAACATCCCCCAAATGGAATGGCTATCCAGTCTTCACACACAATATGCATTTTAATAGAAAAAAAAACATATGTATATTTTGATCGCATAATTGACAAACACAAAATAGTATACAATCAAACAACATCTATTAAGGCGTCTTTCAGATGAGCATCAGCTAAAGAAATATTACTCACAAAGTGAATGGCCGACCACATATCAACGGCACAACAGTATATTGAAATCAAAACGCAATTCAAACAACATACCACCTCATCAGCTAAAACTAGAATTACATCCGCTCTCGCCAAACCTAAAAACTTGATGAAACAAGAATCTTAAAACTAATAAATAAGACGGACTTCAACTAATTACGCAAGTGATTTCAAGTAGTTTTAAGACTCATATATTTGTAAGTCACGTTTGAGAAATCAAATCTGCGTTCTCAAACAATAACGTAAAACACAAGAAAAAACACTTAAAAAAGTAAGACAAATATAGAAAAAAAATTTTTTTATCATATCAATTACAAACATATTGTTTGCTGCAAAACAAAACTAAACATAGGTGAAGTATCCAATGACGTAATTTGAAGTACAAAAGGAGTAACAAGTTACTTATAAAAATAGAAAAACAAAATGGATTATTACAAACTTAAATAGGATTAAACTTAAACTAATTACACCAATCAACTTAACATGAAGCAACTCCTGATACTTTACAGGCCGCCGCACGCGCACCTTAACAGGTGCTTGCTTGGCAGCCTCGTAGGCTTCTAAAAGTTATTTTTCTGTATGCATATGCATATGCATTTGTATAGCATACAACCTTTAATTTAATAGCCATACAACCTAATAGCCAGAAACGCCTTCCCAATTTACTGAACCCTGCTTATATGTAGTTTATAATTGTTGTGTCTTTTTTTTACGTTTAAGTTGTAATTTGATGTTAGAATTTATTACAATGGATGCAAGTTTTAACTCAATTTGATCACATAGGATTTGAATTGTAAAAAGGTCTTCTGCTGACATTATTAGTATGTATTGTGCTTTAGATTAAATTATTGTGTTTTAGTGTATTTTGTTCCTTTGTTTTTGGGTTTCTTTGTTCTGTAAGTAGTTAAATACCCTCCACACTCTACACCTACCTTGCACTCACAACTACAATGGACTCACACCAAGAATTATCAGCGGGTTCCCCTATTTCCTACGACTTTTTAGATCCAGACTGGTGTTTTAAAAGATACCTTACAAAGGATGCTCTTCATTCAATTGAAACTGGCAAAGGTGCTGCTTACTTTGTTCCTGATGGCTTTACTCCTATACTCATTCCTAACTCACAAAGCTACCTGTTGGATGGGAATTCTGCACAACTGCCAAGACCTCAACCAATATCCTTCACATTGGATCAATGTAAAGTTCCCGGCTATATTTTGAAGAGTTTACGGAAAGACACTACGTCTACTGAACGTACTCCGAGACCCCCTAATGCTTTTATATTATACAGGAAGGAAAAACATGCTACATTATTAAAGTCTAACCCTTCCATCAATAATTCACAAGTTTCCAAGCTGGTTGGAGAGATGTGGAGAAACGAATCAAAGGAAGTACGTATGCGATATTTTAAGATGTCTGAGTTTTATAAAGCACAACATCAGAAAATGTACCCTGGTTATAAGTACCAGCCTAGAAAGAATAAAGTTAAAAGATAATAATGGTAATATTTATGGTATTTAATAAAGAATTTTGAAATTGATTTAAATATGCTTCTTAGAGTTACATTCACTGAAGATTATAATGTAATATTTTGTGTACCCCATTTGCGTTGAGTTATTCTATAGTAATTATTGTGTGTTCTATTAACGATGTATTGCGATTTATATCTGTTATGCTAACATAACGTAGTTCTAAGCACTGTAATGCCATACTGTTTTAGAGGGTGATGCTTCCTAAAATCTCCTTACATAAAGTAATACATGGATTTTACTGCCCTGATTCTATCGAAATATGCTGTTTTTTTTATTCGTTTTTATTTATTTTCAATAATTTTTTTGTAATATAAATGTATAGTCTTTCTCCTTTGTTTTCTCTCGTTCGTTTCCATGTTATCCAAATATGTTTGTTTGGCCGATAGTCTGTCTTTTGGGAAAAAATGCGCATTTTGTGTTTTTAAAAGCAGTTTAGAATAGTTTTAAACAAAAAAAGCAATCGTTTTGCCAACATAACGATATCATCATTTAGCAACAACTATAATGTTCAAATTATAACCTGATATTGAGTATATAGTAAATTAATAAATTAGAATTGTTTTACCTATTAAACGTGATCATTTGTACAGTTTTTCTCAACACATTCACTACAAATATTTCACTGACATCAATCGCTAGGCGCTTTAATATAATTTTTTTGAAAACTGGAAACATATTTTTAAGTTAAAATAAAATAACTTGCTCAAAAATATCTAACAGTAGTTGGCAAATAAATAAATAAAAGTTCTTCTTACCCGGATAGACATTTTGATGGAATTATCAGTTACCTTAAAATGCGTTAACCAAATTAACGTCAAACTAGTATAATACATCGCAATAACCATTTAACATATTTCTTCTAGGTAGTAATGAATTACTTCAGTTGACAAAATTTGTGCACTGTGGCTTTCATGTCAATTTTAGTTTTTTCATTCCTATCGGTTTGCTCATACCATATTTTAAA
